# Psychosocial stress and microRNA expression profiles in myometrial tissue of women undergoing surgical treatment for uterine fibroids

**DOI:** 10.21203/rs.3.rs-3373251/v1

**Published:** 2023-09-21

**Authors:** Christian K. Dye, Haotian Wu, Brianna VanNoy, Stephanie Calluori, Cherie Q. Marfori, Andrea A. Baccarelli, Ami R. Zota

**Affiliations:** Department of Environmental Health Sciences, Columbia University; Department of Environmental Health Sciences, Columbia University; Ohio State University College of Medicine; Department of Environmental Health Sciences, Columbia University; Minimally Invasive Gynecologic Surgery, Inova Health Systems; Department of Environmental Health Sciences, Columbia University; Department of Environmental Health Sciences, Columbia University

**Keywords:** Epigenetics, psychosocial stress, fibroids, myometrium, women’s health, health disparity, gynecology

## Abstract

Uterine leiomyomas (fibroids) are the most common non-cancerous tumor affecting women. Psychosocial stress is associated with fibroid risk and severity. The relationship between psychosocial stress and fibroid pathogenesis may involve alterations in microRNAs (miRNAs) although this has yet to be examined. We investigated associations between two psychosocial stress measures, a composite measure of recent stressful life events and perceived social status, with expression levels of 401 miRNAs in myometrium (n = 20) and fibroids (n = 44; 20 matched between tissues) from pre-menopausal women who underwent surgery for fibroid treatment. We used linear regressions to identify psychosocial stressors associated with miRNAs, adjusting for covariates (age, body mass index, and race/ethnicity). Psychosocial stressors were modeled as ordinal variables and results were considered statistically significant if the overall variable significant was below false discovery threshold (FDR < 0.10) and showed a monotonic dose-response (nominal p-trend < 0.05). In the myometrium, 16 miRNAs were significantly associated with total stressful events and two miRNAs were associated with perceived social status. No fibroid miRNAs were associated with either stress measure. Pathway analyses revealed miRNA-mRNA targets were significantly enriched (FDR < 0.05) in pathways relevant to cancer/tumor development. Of the 74 differentially expressed miRNAs between myometrium and fibroids (*p* < 0.05), miR-27a-5p was also associated with stress exposure. Our pilot analysis suggests that psychosocial stress is associated with changes in myometrium miRNAs, and thus, plays a role in the pathogenesis of fibroids from healthy myometrium.

## Introduction

Uterine leiomyoma (fibroids), arising from the smooth muscle cells of the uterus (*i.e*., myometrium), have an estimated incidence of nearly 70% in women during their reproductive years [1]. Although these tumors are non-cancerous, fibroids can result in a range of adverse health outcomes, including common symptoms such as pelvic pain, heavy menstruation, and depression and more severe reproductive health effects, including infertility and pregnancy complications [2, 3]. Mounting evidence suggests the pathogenesis of fibroids involves ovarian hormones, including estrogen and progesterone [4–7]. However, there has been less attention to the social and structural factors that may drive hormonal disruptions [8]. The link between sex hormones and fibroids may arise, in part, from chronic exposure to common psychosocial stressors [9, 10], stress factors that have been associated with both progesterone and estrogen changes [11, 12]. This is especially concerning given the disproportionate burden of fibroids among Black women. For instance, Black women exhibit both a higher incidence of fibroids, reaching more than 80% by age 50, and more severe symptoms compared to White women [13]. The racial disparity in fibroids may arise from increased prevalence of high levels of psychosocial stressors pervasive among Black women [9, 14]. Indeed, perceived racism in Black women has been associated with an increased risk for fibroids [15], which likely operates through discrimination-induced stress responses [16]. Additionally, our group previously reported an association between adverse childhood experiences (ACEs) and fibroid symptom severity. In this study population, ACEs were significantly more prevalent in Black women compared to non-Black women [17]. Although some studies have linked measures of psychosocial stress to risk and severity of fibroids, the potential underlying biological mechanisms that may explain psychosocial stress-induced fibroid formation in at-risk populations remains poorly understood.

MicroRNAs (miRNAs) are small, highly conserved non-coding RNAs involved in post-transcriptional and translational gene regulation [18]. Accumulating evidence has focused on mechanisms operating gene-environment interactions, specifically those molecular mechanisms that are responsive to environmental cues and guide gene expression, and thus may have a role in disease [19, 20]. In addition to their role in uterine development and function [21], miRNAs have been found to play a role in tumorigenesis in reproductive organs, including involvement in fibroid formation from the myometrium [22, 23], and miRNAs have been associated with psychosocial stress responses [24]. Given that miRNAs are responsive to environmental stressors and are fundamental to uterine biology, the relationship between psychosocial stress and fibroid pathogenesis may involve alterations in miRNAs in the myometrium. However, to our knowledge, the role of miRNAs in psychosocial stress-associated fibroid has yet to be investigated. To this end, from women enrolled in the Fibroids, Observational Research on Genes and the Environment (FORGE) study, we conducted a preliminary investigation to examine whether measures of psychosocial stress exposures were associated with myometrium- and fibroid-specific miRNAs, and whether the miRNAs associated with stress exposure were involved in functionally relevant gene networks important to fibroid pathogenesis.

## Methods

### Study population

Our study included participants from the FORGE study, which recruited eligible women for enrollment in 2014–2017 under the inclusion criteria that they were English-speaking, non-pregnant, pre-menopausal adult women (≥ 18 years) who were seeking access to care for symptomatic fibroid management and intened to undergo hysterectomy or myomectomy at hospitals affiliated with the George Washington University (n = 45) [25]. From the total enrollment, 44 participants had data available for psychosocial stressors, clinical and demographic information, and miRNA data collected from either fibroids or myometrium. Of the 44 participants, 20 participants, who underwent a hysterectomy, had miRNA data in myometrium and fibroids, and the remaining 24 participants had miRNA data collected only from fibroids. Participants were primarily Black, college educated, and privately insured at enrollment [25, 26]. The George Washington University Institutional Review Board approved the protocols for the study. All participants provided written informed consent prior to enrollment.

### Data collection

All clinical and demographic data were abstracted from participants’ medical records, including race/ethnicity, age, and body mass index (BMI; kg/m^2^). In order to assess psychosocial stress from recent stressful life events, an interview-administered questionnaire was used consisting of the following ten questions (yes/no responses) regarding stressful life events that happened within the last 12 months, including: 1) a family member was very sick and had to go into the hospital; 2) I got separated or divorced from my husband or partner; 3) I moved to a new address; 4) my husband or partner lost their job; 5) I lost my job even though I wanted to go on working; 6) I argued with my husband or partner more than usual; 7) I had a lot of bills I couldn’t pay; 8) I was in a physical fight; 9) My husband or partner had serious legal problems; and 10) someone very close to me had a problem with drinking or drugs. Given multiple, cumulative stressors may act as better indicators of health than individual stressors [27], we created a cumulative score (recent stressful life events score) from the 10 binary life event questions and converted it into an ordinal categorical variable for analyses (no stress exposure, 0; low stress, 1; medium stress, 2; high stress ≥ 3). Finally, in order to capture perceived social stress as a consequence of social status, we used the social hierarchy ladder response derived from the MacArthur scale [28]. A lower ladder response reflected low perceived social status (indirect indicator of high social stress vulnerability) and a high score indicating high perceived social status (low social stress vulnerability). The ladder response variable was coded as a 3-level ordinal categorical variable for analyses (ladder response: low social standing score, 1–3; mid social standing score, 4–7; high social standing score, 8–10).

### MiRNA collection and data processing

Fibroid tissues were collected from 45 participants following hysterectomy or myomectomy procedures, snap frozen in liquid nitrogen and stored until analysis. Paired myometrium samples were collected from hysterectomy participants only (n = 20). Pathology reports were used to confirm that tumors were non-cancerous. For miRNA expression analysis, frozen tissue samples were incubated with RNAlater-ICE Frozen Tissue Transition Solution (Thermo Fisher Scientific, Waltham, MA, USA), homogenized with a TissueRuptor (Qiagen, Germantown Road, MD, USA), then RNA was purified with the miRCURY RNA Isolation Tissue Kit (SelectScience, Bath, UK). RNA samples were cleaned and selected for small RNAs using the RNA Clean & Concentrator Kit (Zymo Research, Irvine, CA, USA). From 100 ng of RNA, we quantified expression of 754 miRNAs and 4 controls (ath-miR159a, RNU48, RNU44, and U6) using the TaqMan^™^ OpenArray^™^ Real-Time PCR Master Mix (Thermo Fisher) on the QuantStudio 12K Flex Real-Time PCR System (Thermo Fisher). The quantification cycle values (C_q_) used in the real-time qPCR represent the PCR amplification cycle in which the fluorescence of the target miRNA exceeded background fluorescence, which is used to quantify the relative expression of each miRNA. Lower C_q_ values translate to higher levels of the target miRNA in a sample.

Raw miRNA data was processed by keeping only miRNAs with an amplification score ≥ 1.1, C_q_ confidence ≥ 0.8, and miRNA with a C_q_ value ≥ control probe U6 expression (4.4099); C_q_ values that did not pass this first check were set to missing. Next, we normalized each miRNA relative expression by subtracting the C_q_ from the global mean of control miRNAs from the targeted miRNA C_q_ value (ΔC_q_). From normalized miRNAs, we then removed all control miRNAs, miRNAs that were tagged in other species (*i.e.*, mouse and rat), and any miRNA that was not observed in ≥ 70% of participants for both myometrium and fibroids. In total, 401 miRNAs were retained for analysis. Next, due to inherent limitations of qPCR technologies, wherein particular miRNAs may be expressed below the limit of quantification [29], we winsorized the remaining missing values (target miRNA C_q_ mean + 3 standard deviations).

### Statistical analyses

The psychosocial stressor variables were modeled as both continuous and ordinal variables. To test the association between measures of psychosocial stress and miRNA expression in myometrial tissue (n = 20), we first modeled recent stressful life events score (ordinal categorical) or the ladder response (ordinal categorical) as the independent variable and miRNA expression as the dependent variable (continuous) while adjusting for *a priori*-selected covariates. *P*-values were calculated using likelihood ratio (LR) tests. The covariates included age, BMI (kg/m^2^), and race (Black or white/other). To adjust for multiple comparisons, we used the Benjamini-Hochberg method at a false-discovery rate (FDR) *p* < 0.10. In addition, we calculated p-trend using linear recent stressful life events score and ladder response variables. Associations were considered to be significant if the overall psychosocial stress variable was below the FDR cutoff (< 0.10) and a linear association was observed (*p*-trend < 0.05). We reported the β-coefficient (95% confidence interval [CI]) for the linear regression results to show the relative change in miRNA expression associated with either the recent stressful life events score or the ladder response. We used the same approach to test associations between stress exposures and miRNAs in fibroids (n = 44).

In a secondary analysis, we used linear regression analyses to test whether 9 out of the 10 individual stressful life events (‘husband or partner had serious legal problems’ removed due to no participants having answered ‘yes’) were associated with the same miRNAs that were associated with the composite stressful life events score. Our secondary analyses were adjusted for *a priori*-selected covariates (age, race, and BMI) and considered significant at *p* < 0.05. Finally, we used generalized estimating equations (GEE) to confirm the 74 differentially expressed (DE) miRNAs between myometrium and fibroid, identified by our group previously [6]. We then compared the DE miRNA list with the list of miRNAs that were associated with the recent stressful life events score to determine whether psychosocial stress was linked to miRNAs that may underlie tissue-specific miRNA profiles.

### Gene ontology analyses

We used the publicly available DIANA-miRPath-v4.0 webserver for functional analysis of miRNAs, which allowed us to perform gene ontology (GO) analysis of biological processes and Kyoto Encyclopedia of Genes and Genomes (KEGG) pathway analysis of potential miRNA-mRNA interactions [30]. DIANA-miRPath integrates a resampling without replacement approach from annotated miRNAs to produce empirical *p*-values as well as controls for errors due to multiple comparisons using the Benjamini-Hochberg method in order to determine mRNA targets of miRNAs. Here, we only considered experimentally validated mRNA targets. Significantly enriched GO processes and KEGG pathways were considered based on an FDR *p*-value < 0.05.

## Results

### Participant characteristics

Clinical, demographic, and psychosocial information from participants with miRNA data collected from both myometrium and fibroids (n = 20) can be found in Table 1. Overall, the mean age (mean [IQR]) of participants was 44.2 (39.2, 49.9) years, 16 participants (80.0%) were Black, and mean BMI was 34.5 (27.3, 41.8) kg/m^2^. The most observed recent stressful life event was, “a family member being sick or hospitalized” (11 [55%]), followed by “many bills that could not be paid” (8 [40.0%]). Most women (15 [75.0%]) viewed themselves in the middle social status group (scored between 4 to 7 out of 10) using the MacArthur subjective social status scale (*i.e*., ladder response). Further, we grouped participants by the amount of perceived psychosocial stress exposure using the recent stressful life events score (score range 0 to 6), where those with exposure to < 2 acute stressors were considered as low stressors (n = 9), and mid-high stressors (n = 11) were those exposed to ≥ 2 acute stressors (Table 1). For the sub-analyses, we included all 44 participants who had miRNA data quantified in fibroids, complete clinical demographic data, and psychosocial stressor information, which can be found in Online Resource 1.

### Psychosocial stress is associated with miRNA expression in the myometrium

In linear regression analyses, we found 16 of 401 miRNAs in the myometrium were significantly associated with the recent stressful life events score after adjustment for multiple comparisons (FDR *p* < 0.10) (Table 2). All 16 miRNA showed a negative association between the recent stressful life events score and miRNA C_q_ values, in which an increasing score is associated with a decreasing miRNA C_q_ value (*i.e*., higher miRNA expression). Two miRNAs, miR-1275 and miR-487a-3p, were significantly associated with the ladder response (Table 3), and both displayed a positive relationship with the ladder response, in which a higher perceived social status was associated with increased miRNA C_q_ values (*i.e*., lower miRNA expression). In contrast, we found no statistically significant associations between fibroid miRNAs and either stress measure after correcting for multiple comparisons (Online Resource 2).

**Figure 1:**
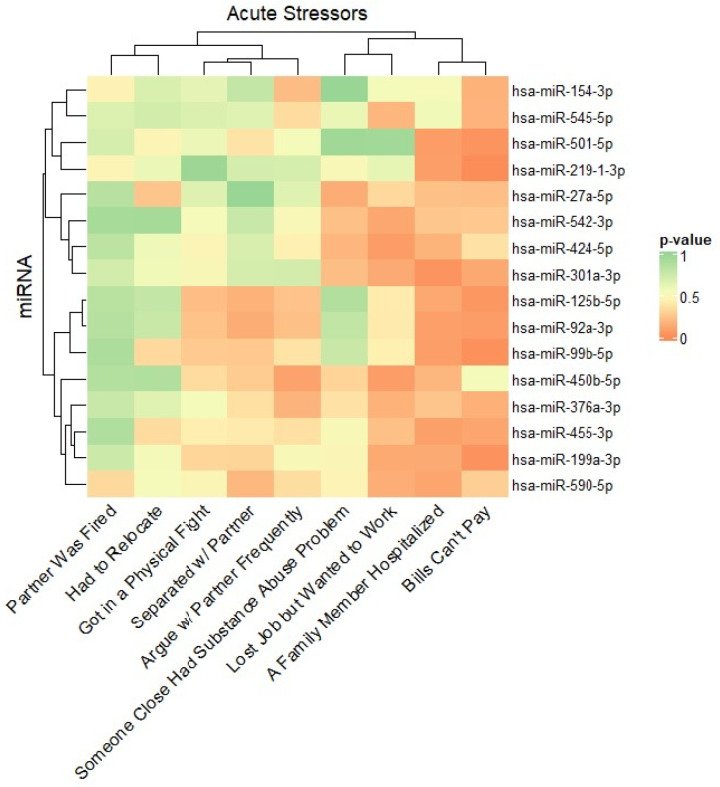
Recent stressful life events score -associated miRNAs in myometrium and individual acute stressors Heatmap produced by Euclidean distance and average-linkage hierarchical clustering of linear regression *p*-values for the association between the 16 recent stressful life events score-associated miRNAs within the myometrium and 9 of the individual acute stressors culminating the score. Significant associations (*p*<0.05) are colored orange.

Given the recent stressful life events score is a composite measure of psychosocial stress across ten individual stressful life events, in a secondary analysis we investigated whether specific life events were associated with the cumulative score-associated miRNAs in myometrium. Here, we found a significant negative association (*p* < 0.05) between ‘Bills Can’t Pay” and 5 myometrium-derived miRNAs (Fig. 1), including miR-219–1-3p, miR-99b-5p, miR-199a-3p, miR-501–5p, and miR-125b-5p. Likewise, we observed a significant negative association between those that have had “A Family Member Hospitalized” and expression of one miRNA, miR-301a-3p (Fig. 1). Although non-significant, a negative relationship was trending (*p <* 0.10) between miRNA expression and “Bills Can’t Pay” and miR-92a-3p; “Lost Job but Wanted to Work” with miR-424–5p and miR-450b-5p; and between “A Family Member Hospitalized” with miR-501–5p, miR-219–1-3p, miR-99b-5p, miR92a-3p, and miR-455–3p (Fig. 1). These results indicate lower C_q_ values (*i.e*., higher miRNA expression) in myometrium from those with exposure to specific acute stressors.

### Psychosocial stress-associated miRNAs enriched at functionally relevant biological pathways

Using the online toolkit DIANA-miRPath, the 16 recent stressful life event score-associated miRNAs in myometrium were significantly enriched at biological processes that were associated with as many as all 16 miRNAs and up to 537 predicted mRNA targets (Online Resource 3). Of the top 50 significant biological processes (FDR *p* < 0.05), these miRNAs were linked to mRNAs that were involved in processes related to cancer, including the cell cycle, cell division, and apoptosis, and viral processes, and transcriptional regulation. KEGG pathway analysis revealed the recent stressful life event score-associated miRNAs were targeted to as many as 250 predicted mRNA targets culminating all 16 miRNAs (Online Resource 4). Of the Top 50 significantly enriched KEGG pathways (FDR *p* < 0.05), miRNA-mRNA targets were involved in pathways potentially relevant to fibroid development, including protein processing in the endoplasmic reticulum (ER), proteoglycans in cancer, cell cycle, p53 signaling pathways, endometrial cancers, and more (Online Resource 4). There were no predicted gene targets and associated processes for the two miRNAs associated with the ladder response (data not shown).

### Psychosocial stress-associated miRNAs and tissue-specific expression patterns

As our results suggest that psychosocial stress is associated with miRNAs in the myometrium but not fibroids, we posited that dysregulated miRNAs in the myometrium may be informative targets for understanding their role in the pathogenesis of fibroids from health myometrium. To this end, we investigated whether recent stressful life events score-associated miRNA in myometrium displayed tissue-specific expression patterns between myometrium and fibroids. Here, using 74 miRNAs previously identified by our group that were differentially expressed between myometrium and fibroids (Online Resource 5) [25], we found one miRNA, miR-27a-5p, was significantly differentially expressed between tissue types (β-coefficient [95% CI]: 1.25 [0.37, 2.14], *p* = 0.01) and significantly associated with the recent stressful life events score in myometrium (−0.93 [−1.42, −0.43]).

## Discussion

In this pilot cross-sectional study of pre-menopausal women, we found a cumulative measure of recent stressful life events and a measure of perceived social standing were significantly associated with expression of select miRNAs in the myometrium, but not in fibroid tumors. We found that one miRNA, miR-27a-5p, was both associated with our composite stressful life event measure and differentially expressed between fibroids and myometrium. These results suggest miRNAs in the myometrium may be altered in response to adverse social conditions, such as exposure to common stressors, and may help us understand the disproportionate burden of fibroids observed in Black women, who often are exposed to a greater range, duration, and intensity of psychosocial stressors [8]. Taken together, we propose that healthy myometrium may become primed to a transitionary state in fibroid development via miRNAs that are potentially dysregulated by acute stressors.

Common stressors can have short- and long-term effects on physical health, and miRNAs may provide a key regulatory mechanism by which stress may contribute to fibroid pathogenesis [9, 24, 31, 32]. In our analysis of myometrial tissue, we observed 16 miRNAs that were significantly associated with a cumulative measure of exposure to common acute stressors, wherein greater stress exposures were associated with higher levels of miRNA expression. In agreement with these results, two miRNAs were associated with one’s perceived social standing (*i.e*., ladder response), as an indirect measure of psychosocial stress, wherein high social standing, and potentially less stress exposure, was associated with lower miRNA expression. However, we did not observe any significant associations between psychosocial stress factors, such as a composite score or through perceived social standing, on fibroid miRNA expression. This may reflect the microenvironment of a diseased tissue (*i.e*., fibroids) exhibiting an already distressed state that is less sensitive to modification caused by additional stressors, which has been observed in other types of cancers as compared to healthy tissues [33].

MiRNAs play an essential role in post-transcriptional gene regulation with each miRNA having the capability to regulate multiple gene targets and genes having multiple miRNAs [34, 35]. Due to the inherent regulatory role of miRNAs, we investigated the predicted gene targets of psychosocial stress-associated miRNAs in the myometrium to allude to the functional role of miRNA via their predicted mRNA targets. The most significant GO biological process was enriched at viral processes, and recent evidence suggests reproductive tract infections and reparative mechanisms that occur in response may initiate fibroid development [36]. Additionally, of the most significant GO biological processes enriched by miRNA-mRNA targets, we found several processes related to cancer. Fibroids are benign tumors that can be characterized by a life cycle similar to cancers, which starts with aberrant cell proliferation and eventually ends in cell death, a continual process that, in addition to extracellular matrix deposition, forms a fibrotic tumor [37]. These processes are likely guided, in part, by regulatory mechanisms of miRNAs, that when dysregulated, as can occur in psychosocial stress-related exposure, may induce cancer-related pathways in the myometrium that contribute to fibroid development [38]. Corroborating these findings, we found the most significant KEGG pathways enriched by social stress-associated miRNA-mRNA targets were at pathways relevant to reproductive tumorigenesis, including endometrial cancers, which have been observed concurrently in those with fibroids [39], and p53 signaling, a fundamental signaling pathway required for fibroid growth [40], as well as pathways in the endoplasmic reticulum (ER) and proteoglycans. Indeed, fibroids exhibit a higher abundance and swelling of the ER [37] and contain different composition of proteoglycans within extracellular matrix deposits [41].

We observed that miR-27a-5p was differentially expressed between myometrium and fibroids, and significantly associated with psychosocial stress exposure, which suggests that miR-27a-5p may be dysregulated by cumulative stress exposure and potentially contribute to the transformation of healthy myometrium into fibroids. Notably, miR-27a is a novel biomarker for solid tumors and has been considered as a therapeutic target for a variety of cancers [42, 43] and may be involved in female infertility [44]. Consistent with our findings, previous studies have identified miR-27a as being differentially expressed between myometrium and adjacent fibroids [45]. Taken together, exposure to psychosocial stress may lead to long-term changes in miRNA expression within the myometrium, and dysregulation of miRNAs in myometrium may have a regulatory role for functionally relevant genes involved in pathways necessary for the transformation of healthy tissue into fibrotic states characteristic of fibroids.

Given that fibroids arise from the myometrium, we propose that miRNAs, in addition to other epigenetic modifications, may prime healthy myometrial tissue towards fibroids, a process that is affected by accumulating exposure to adverse environmental influences, including psychosocial stressors. This is especially important for minoritized populations, where exposure to adverse psychosocial conditions is quite common throughout the life-course. Indeed, previous evidence by our group demonstrated that adverse childhood experiences (ACEs) were associated with greater severity of these fibroids, and ACEs were more prevalent among Black women than non-Black women, further supporting the relevance of our current work to understanding racial disparities in fibroids [17]. We posit that as an individual acquires specific adverse “hits”, or risk factors (*i.e*., race, obesity, diet, age, parity, *etc*.), throughout the life-course, these hits can drive pathological processes within the myometrium that may initiate the development of fibroids [46], a process that may be guided by epigenetic mechanisms that can affect the myometrial stem cell pool, their self-renewal states, and their final differentiated state in myometrium tissues [47]. These epigenetic changes in myometrial tissues are thus primed to a pro-fibroid phenotype that may readily respond to adverse environmental conditions that contribute to fibroid development. Recent evidence found epigenetic mechanisms may underlie the racial differences in fibroid disparities and primed myometrial stem cells are expanded in Black women [48, 49]. Taken together, it is plausible that the disproportionate prevalence of fibroids in underserved communities, including Black women, may arise from epigenetic priming of progenitor myometrial cells and otherwise healthy myometrium to a pro-fibroid state early during development that is amenable to the effects of environmental exposures (*i.e*., psychosocial stressors), that, when accumulating over time, may readily transition to fibroids.

This study provides evidence for miRNAs as a potential epigenetic mechanism involved in psychosocial stress-associated fibroid development. However, as this is a pilot study, results should be interpreted with caution. In our analyses, we had a modest sample size of 20 participants and higher likelihood of spurious results. Our analyses were primarily focused on two types of psychosocial stressors, acute life events and stress associated with social standing (*i.e*., ladder response), although there are many types of psychosocial stressors (*e.g*., structural racism, ACEs, *etc*.), that were not included in this study. Thus, there could be residual confounding from these unmeasured confounders. Likewise, all participants were enrolled based on the criteria of seeking surgical treatment for fibroids, and our prior work has shown that the process of seeking medical treatment for fibroids can be a unique and important source of stress [50, 51]. As this study included participants who underwent surgical treatment for fibroids, these results may not be generalizable to women with asymptomatic fibroids. Because myometrium and fibroid tissue contain heterogenous cell populations, we were unable to ascertain the cell type-specific expression patterns of miRNAs; however, myometrium and fibroids predominantly contain smooth muscle cells. Our *in-silico* approach sought to determine mRNA targets of social stress-associated miRNAs to infer biological functionality, although individual miRNAs can target multiple genes and mRNA targets differ by cell type and tissue type [52]. The enrichment of biological functions and pathways relevant to tumorigenesis at an FDR *p*-value < 0.05 provide confidence in our analysis. Future studies should seek to expand on our findings in larger, longitudinal cohorts of diverse populations of women and include a diversity of psychosocial stressors in independent and composite analyses. Nonetheless, our preliminary study provides compelling evidence for the effects of psychosocial stress exposure from recent life events and social status-associated stress on miRNA signatures in otherwise healthy myometrium tissue that may contribute to fibroid development that can be used as a framework for future research.

In conclusion, our study suggests cumulative exposure to typical acute stressors may contribute to fibroid progression and pathogenesis through dysregulation of miRNAs. Our findings may have important implications for understanding and ultimately mitigating the disproportionate burden of fibroids in Black women since this work suggests the priming of myometrial tissues to pro-fibroid states may be susceptible to social and environmental exposures, including psychosocial stress.

## Figures and Tables

**Table 1.** Participant characteristics of FORGE participants with both myometrium and fibroid miRNA data available

Variables	Low Stressor Group (<2 Acute Stressors; n=9)	Medium to High Stressor Group (n=11)	Overall (n=20)

Age (years)	47.5 (46.3, 51.8)	41.5 (37.9, 46.6)	44.2 (39.2, 49.9)
Black/African American Race, n (%)	5 (55.5%)	11 (100.0%)	16 (80.0%)
BMI (kg/m^2)	32.1 (28.5, 39.8)	35.6 (27.1,42.8)	34.5 (27.3, 41.8)
Reported Acute Stressors, n (%)			
Family member sick and hospitalized	2 (22.2%)	9 (81.8%)	11 (55.0%)
Separated or divorced from partner	-	2 (18.2%)	2 (10.0%)
Had to relocate	1 (11.1%)	2 (18.2%)	3 (15.0%)
Partner lost job	1 (11.1%)	1 (9.1%)	2 (10.0%)
Lost job but still wanted to work	-	3 (27.3%)	3 (15.0%)
Argued w/ partner more than usual	-	4 (36.4%)	4 (20.0%)
Lots of bills I couldnť pay	1 (11.1%)	7 (63.6%)	8 (40.0%)
In a physical fight	-	1 (9.1%)	1 (5.0%)
Partner had serious legal problems	-	-	-
Someone close had drinking/drug problem	-	3 (27.3%)	3 (15.0%)
Ladder Response, n (%)			
Low social status (score 1–3)	-	2 (18.2%)	2 (10.0%)
Mid social status (score 4–7)	7 (77.8%)	8 (72.7%)	15 (75.0%)
High social status (score 8–10)	2 (22.2%)	1 (9.1%)	3 (15.0%)

Continuous variables: mean (1st, 3rd Quartile); categorical variables: count (%). BMI: body mass index

Acute stressors determined by questionnaires as psychosocial stressors occurring within the past 12 months (yes/no)

Participants classified as Low Stressors based on a recent stressful life events score < 2; or Medium to High Stressors based on a recent stressful life events score ≥ 2

Recent stressful life events score (ordinal categorical) calculated as a cumulative score using the sum of acute stressors (binary); score of 0 (no acute stressors) to 10 (high acute stressors)

Ladder response is derived from the MacArthur subjective social status scale - perceived rank relative to other groups

**Table 2:** Recent stressful life events score-associated miRNA in myometrium

miRNAs	β-Coefficient (95% CI)	LR Test FDR *p*-value	Linear Regression *p*-trends
miR-125b-5p	−0.71 (−1.23, −0.19)	0.1	0.01
miR-154-3p	−0.88 (−1.39, −0.38)	0.04	0.002
miR-199a-3p	−0.67 (−1.05, −0.30)	0.1	0.002
miR-219-1-3p	−0.81 (−1.32, −0.31)	0.07	0.004
miR-27a-5p	−1.15 (−2.00, −0.30)	0.04	0.01
miR-301a-3p	−0.93 (−1.42, −0.43)	0.07	0.001
miR-376a-3p	−0.68 (−1.04, −0.33)	0.06	0.001
miR-424-5p	−1.07 (−1.65, −0.49)	0.07	0.001
miR-450b-5p	−1.11 (−1.71, −0.50)	0.1	0.001
miR-455-3p	−1.05 (−1.78, −0.32)	0.1	0.01
miR-501-5p	−0.87 (−1.66, −0.09)	0.07	0.03
miR-542-3p	−1.28 (−2.02, −0.55)	0.1	0.002
miR-545-5p	−1.52 (−2.21, −0.82)	0.05	0.0003
miR-590-5p	−0.62 (−1.01, −0.24)	0.1	0.004
miR-92a-3p	−0.70 (−1.20, −0.21)	0.1	0.01
miR-99b-5p	−0.88 (−1.51, −0.25)	0.1	0.01

Linear regression *p*-value based on regressing miRNA expression on the recent stressful life events score (ordinal categorical)

Likelihood ratio (LR) test performed between linear regression and a nested linear regression that regressed miRNAs on linear regression covariates

Linear regression adjusted for age, body mass index ((kg/m^2), and race/ethnicity (white/other vs African American)

5p refers to miRNA on the forward position (5’–3’); 3p refers to miRNAs present in reverse position (3’–5’)

Abbrv.: miR- microRNA; FDR- false-discovery rate; CI- confidence interval; LR- likelihood ratio test

Significance taken at an LR Test FDR p-value < 0.10

**Table 3:** Ladder response-associated miRNA in myometrium

miRNAs	β-Coefficient (95% CI)	LR Test FDR *p*-value	Linear Regression *p*-trend
miR-1275	1.77 (0.96, 2.59)	0.03	3.23E-04
miR-487a-3p	1.77 (0.61,2.93)	0.03	0.01

Linear regression *p*-value based on regressing miRNA expression on the ladder response (continuous)

Likelihood ratio (LR) test performed between linear regression and a nested linear regression that regressed miRNAs on linear regression covariates.

Linear regression adjusted for age, body mass index ((kg/m^2), and race/ethnicity (white/other vs African American)

Significance taken at an LR Test FDR p-value < 0.10

## Data Availability

The data that supports findings from this study are available upon reasonable request from ARZ. The data is part of a larger on-going collection for the FORGE study and is currently not publicly available.
